# ﻿*Carpinuswenshanensis* (Betulaceae), a new species from southeast Yunnan, China

**DOI:** 10.3897/phytokeys.260.137709

**Published:** 2025-07-15

**Authors:** Xiao-Long Zhou, Qian-Na An

**Affiliations:** 1 College of Ecology and Environment, Xinjiang University, Urumqi 830046, China Xinjiang University Urumqi China; 2 Key Laboratory of Oasis Ecology, Xinjiang University, Urumqi 830046, China Xinjiang University Urumqi China

**Keywords:** hornbeam species, morphological traits, phylogenetic position, southeast Yunnan

## Abstract

*Carpinuswenshanensis* Xiaolong Zhou, a newly discovered hornbeam species endemic to Wenshan Prefecture in southeast Yunnan, China, is described and illustrated. This study employs an integrative taxonomic approach, combining morphological comparison, biogeographic distribution analysis, and phylogenetic reconstruction based on nuclear ITS sequences, to establish *C.wenshanensis* as a distinct species sister to *C.tschonoskii*. *Carpinuswenshanensis* is diagnostically characterized by six key morphological traits: (1) mucronate-serrate leaf margins (versus setiform serrate in *C.tschonoskii*); (2) serration restricted to the basal half of outer bract margins (versus extending across both basal and apical halves); (3) reduced serration number on outer bract margins (1–3 serrations per margin compared to 4–7); (4) nearly glabrous petioles and annual branchlets (versus sparsely or densely pubescent); (5) longer petioles (1.0–1.7 cm versus 0.6–1.3 cm); and (6) a variable leaf base shape (rounded, subrounded, cordate, or subcordate versus rounded-cuneate or cuneate). Additionally, population-level ITS sequence comparison identified three fixed nucleotide polymorphisms that unequivocally differentiate *C.wenshanensis* from its closest relative. Collectively, these findings provide robust evidence for recognizing *C.wenshanensis* as a novel species within the genus *Carpinus*.

## ﻿Introduction

The hornbeam genus *Carpinus* L. (Betulaceae, subfamily Coryloideae) is the most species-rich group within its subfamily, comprising approximately 46 recognized species extending across three of seven continents in the world (Li and Skvortsov 1999; Holstein and Weigend 2017; Lu et al. 2017, 2018, 2024). Current hornbeam taxonomy relies heavily on morphological traits from bracts, nutlets, leaves, and branchlets, particularly for bract morphology (Li and Skvortsov 1999; Lu et al. 2017, 2018; Lu 2020). For instance, the two primary sections, *Carpinus* and *Distegocarpus*, are distinguished by bracts that either fully or partially envelop the nutlet (Dong et al. 2022), and the extreme form like *C.gigabracteatus* Z.Q.Lu is defined by exceptionally large bracts (Lu 2020).

Within section Carpinus, species are further classified based on the presence or absence of an inflexed basal lobe on the inner bract margin (Li and Skvortsov 1999; Lu 2020). Species exhibiting this lobe include *C.tschonoskii* Maxim. (see the following representative specimens: PE 00021940, PE 00021941, PE 01698545, and PE 00021963 [CVH, https://www.cvh.ac.cn]), *C.putoensis* W.C.Cheng, *C.langaoensis* Z.Q.Lu & J.Q.Liu, *C.mianningensis* Yi, *C.kweichowensis* Hu, *C.tientaiensis* W.C.Cheng, *C.betulus* L., *C.austrobalcanica* D.Lakušić, Kuzmanović, Stevanoski, Schönsw. & Frajman, *C.caroliniana* Walter, *C.tropicalis* (Donn.Sm.) Lundell, *C.laxiflora* (Siebold & Zucc.) Blume, *C.viminea* Wall. ex Lindl., *C.londoniana* H.J.P.Winkl., and *C.fargesii* Franch. (Furlow 1987; Yi 1992; Li and Skvortsov 1999; Holstein and Weigend 2017; Lu et al. 2017; Dong et al. 2022; Kuzmanović et al. 2024; Lu et al. 2024). The presence of a flattened basal lobe on the outer bract margin (whether trilobed or not) further differentiates species such as *C.betulus*, *C.austrobalcanica*, *C.caroliniana*, and *C.tropicalis* from most of Chinese hornbeams. In China, only two species—*C.tientaiensis*, and *C.londoniana*—possess trilobed bracts (i.e. basal lobes on both inner and outer bract margins) (Li and Skvortsov 1999; Lu et al. 2017, 2024).

**Figure 1.**
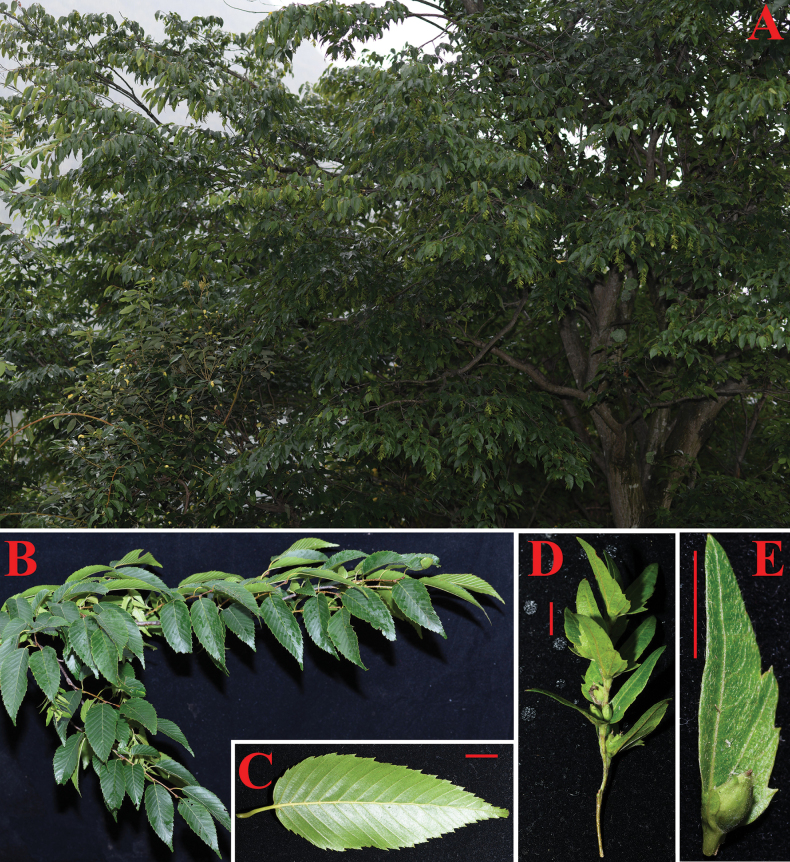
*Carpinuswenshanensis* Xiaolong Zhou. **A.** The mature tree; **B.** Several branchlets with infructescences and leaves; **C.** Leaf; **D.** A representative infructescence; **E.** Nutlet and the bract with a prominent lobe at the base of inner margin. All red bars are one centimeter long.

China harbors nearly three-quarters of global *Carpinus* diversity, with most species endemic to the region and exhibiting narrow distributions (Holstein and Weigend 2017; Lu et al. 2017, 2018, 2024; He et al. 2021; Kuzmanović et al. 2024). Recent extensive field surveys have revealed numerous new species in China (Tong et al. 2014; Lu et al. 2017, 2018; Lu 2020). During fieldwork in Wenshan Prefecture, Yunnan, we documented a distinctive *Carpinus* population (one mature tree and three seedlings) characterized by an inflexed basal lobe on the inner bract margin. This population exhibits distinct morphological differences from each of 12 species (*C.tschonoskii*, *C.langaoensis*, *C.kweichowensis*, *C.betulus*, *C.austrobalcanica*, *C.caroliniana*, *C.tropicalis*, *C.laxiflora*, *C.putoensis*, *C.mianningensis*, *C.viminea*, and *C.fargesii*) in at least three key traits from bract, nutlet, leaf, and annual branchlet (Li and Skvortsov 1999; Kuzmanović et al. 2024; Lu et al. 2024; Fig. 1). Although *C.londoniana* occasionally exhibits only the inner basal lobe (Lu et al. 2024), the Wenshan population differs significantly in bract length, nutlet size, and nutlet pubescence. These distinctions preclude assignment to any described Chinese *Carpinus* species, suggesting a novel species.

Standard Nucleotide BLAST analysis (NCBI) revealed that the ITS sequence fragment of the putative new species is most similar to *C.tschonoskii*, yet distinguished by three fixed nucleotide polymorphisms (Table 1). Morphologically, the putative new species differs from *C.tschonoskii* in mucronate-serrate leaf margins (versus setiform serrate), nearly glabrous petioles and annual branchlets (versus sparsely or densely pubescent), and elongated petioles (Li and Skvortsov 1999).

**Table 1.** Nuclear ITS sequence variations between the two closely related hornbeams (*C.wenshanensis* vs. *C.tschonoskii*). The three fixed nucleotide polymorphisms of *C.wenshanensis* are shown in bold. Variable positions interpreted based on the aligned sequences where polymorphisms occur.

Species (number of downloaded sequences/newly obtained sequences in this study)	Variable positions								
			1	5	5	5	5	5	5
	5	5	5	2	3	3	7	9	9
	6	8	7	5	5	7	7	5	7
*Carpinuswenshanensis* Type 1 (1/0)	**G**	**T**	T	G	C	**T**	C	A	G
*Carpinuswenshanensis* Type 2 (1/0)	**G**	**T**	T	T	G	**T**	C	A	G
*Carpinustschonoskii* Type3 (1/0)	C	C	Y	T	C	G	C	A	G
*Carpinustschonoskii* Type4 (4/0)	C	C	C	T	C	G	C	A	G
*Carpinustschonoskii* Type5 (37/29)	C	C	T	T	C	G	C	A	G
*Carpinustschonoskii* Type6 (1/0)	C	C	T	T	C	G	C	R	G
*Carpinustschonoskii* Type7 (1/0)	C	C	T	T	C	G	Y	A	G
*Carpinustschonoskii* Type8 (2/0)	C	C	T	T	C	G	C	A	T

*Carpinustschonoskii* is a widespread hornbeam species native to China, Japan, and Korean Peninsula. While Li and Skvortsov (1999) synonymized *C.mianningensis* Yi, *C.falcatibrateata* Hu, *C.obovatifolia* Hu, and *C.paohsingensis* W.Y.Hsia under *C.tschonoskii*, recent work by Lu et al. (2017) challenges the inclusion of *C.mianningensis* within this synonymy. Accordingly, we recognized *C.mianningensis* as a distinct species in this study. We note that *C.paohsingensis* has also been maintained as independent species status by Govaerts and Frodin (1998). To evaluate the taxonomic status of the putative new species, we conducted phylogenetic analysis of nuclear ITS sequences to resolve its evolutionary relationship, supplemented by comprehensive morphological comparisons and sequence variation analysis with *C.tschonoskii* and other close relatives.

## ﻿Material and methods

### ﻿Field surveys and collection

Field investigations (in the years of 2023 and 2024) in Wenshan Prefecture, southeastern Yunnan, identified a putative new *Carpinus* species. Preliminary photographic evidence in 2023 indicated taxonomic novelty, and later, we confirmed this through detailed morphological examination in 2024. During August 2024 fieldwork, we documented a population of four individuals: one mature tree (ca.11 m) and three seedlings (0.5–2.5 m). We conducted full morphological characterization, habitat assessment, and preliminary conservation evaluation.

Voucher specimens (X. L. Zhou 2024WS01–2024WS03) and silica-dried leaf samples from the three largest individuals were collected for morphological and molecular analyses. Comparative specimens from putative close relatives were obtained. All vouchers are deposited at XJU Herbarium (Xinjiang University Herbarium).

### ﻿DNA extraction and sequencing

The nuclear ITS (internal transcribed spacer region) region, a core DNA barcode, exhibits high discrimination ability at the species level (CBOL Plant Working Group, 2009). We employed the universal primer pair ITS1a (5′-TCCTCCGCTTATTGATATGC-3′) and ITS4a (5′-GGAAGTAAAAGTCGTAACAAGG-3′), which has been successfully used in *Ostrya* (Lu et al. 2016)—a genus phylogenetically nested within *Carpinus* (Dong et al. 2022)—and has shown robust species-level discrimination when applied to *Carpinus* (Lu et al. 2017, 2018). Given these advantages, we selected this primer pair to amplify this DNA barcode for phylogenetic analysis and sequence variation analysis. Total genomic DNA was extracted using a modified CTAB protocol (Doyle and Doyle 1990) and normalized to 50 ng/µl for PCR amplification. Reactions (50 µL total volume) contained 2 µL DNA template, 5 µL 10 × PCR buffer, 0.8 µL dNTPs (2.5 mM), 2 µL each primer (10 µM), 0.4–0.5 µL *rTaq* polymerase, and ddH_2_O. Amplification conditions were: initial denaturation at 94 °C for 4 min; 38 cycles of 94 °C (40 s), 60 °C (45 s), and 72 °C (2 min); and final extension at 72 °C for 6 min. PCR products were verified via agarose gel electrophoresis, and qualified PCR products were sequenced. From the four sampled individuals, high-quality ITS sequences were obtained only for the mature tree and one seedling. These sequences were deposited in GenBank under accessions PQ492316 (mature tree) and PQ492317 (seedling).

### ﻿Phylogenetic analysis

To elucidate the phylogenetic position of the putative new species, we analyzed ITS sequences from 90 accessions (two newly obtained sequences and 88 downloaded sequences) representing 35 *Carpinus* species—approximately three-quarters of the genus’ known diversity. Sequences were aligned using MEGA 5 (Tamura et al. 2011), yielding a 603-bp alignment. A Neighbor-Joining (NJ) tree was constructed in MEGA version 5.0 with default parameters (Tamura et al. 2011), rooted using four outgroup species (*C.japonica* Blume, *C.fangiana* Hu, *C.rankanensis* Hayata, and *C.cordata* Blume), which form a monophyletic clade different from sect. Carpinus (Dong et al. 2022).

### ﻿Morphological comparison

We performed comprehensive morphological comparison with *C.tschonoskii* and closely related species, following Lu et al. (2024). All leaves from each specimen were measured to capture intraspecific variation and assess diagnostic traits supporting the distinctiveness of the putative new species.

## ﻿Results

### ﻿Phylogenetic position based on ITS sequence variations

**Figure 2.**
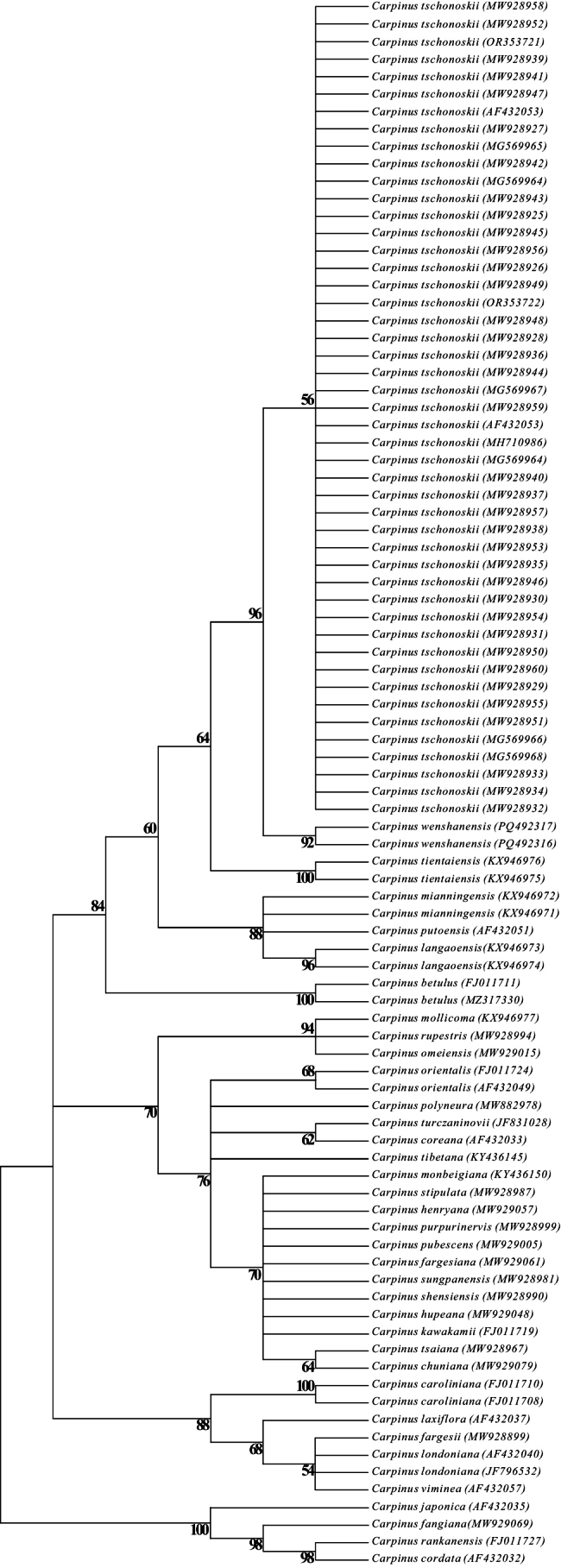
A Neighbor-Joining (NJ) tree based on nuclear ITS sequences of hornbeam species. *Carpinusjaponica*, *C.fangiana*, *C.rankanensis*, and *C.cordata* are outgroups. GenBank accession numbers are presented in brackets.

Phylogenetic analysis of nuclear ITS sequence variations resolved three clades with moderate support (70–90%) within section Carpinus (Figs 1, 2), and their topology aligns with prior studies (Lu et al. 2017, 2018; Dong et al. 2022; Kuzmanović et al. 2024). Hornbeams without the significant basal lobe on the inner bract margin comprised one clade, and those with that lobe were divided into two clades. Based on the phylogenetic tree (Fig. 2), the putative new species and *C.tschonoskii* form a well-supported subclade with a robust support (96%), showing sister relationship between each other. To evaluate intraspecific variation, we sequenced ITS from 32 *C.tschonoskii* individuals across five populations (Shaanxi, Sichuan, Hunan, Hubei), obtaining 29 high-quality sequences (Tables 1, 2). Combined with published data, eight *ITS* types or sequences were identified (Table 1), with all new sequences completely matching the predominant sequence (Type 5 in Table 1) reported in a prior population-level study (Dong et al. 2022). This complete consistency (no sequence variation) across geographically distant populations confirms the reliability of using ITS sequences for species identification. Only one representative sequence was deposited in GenBank (PV151470). Critically, the putative new species differs from all identified *C.tschonoskii* types or sequences by three fixed nucleotide substitutions (Table 1), providing unambiguous molecular evidence for its distinctiveness.

**Table 2.** Specimens of *C.tschonoskii* used for morphological comparison. Type specimens of its synonyms (*C.falcatibrateata*, *C.obovatifolia*, and *C.paohsingensis*) are also included.

Collector	Barcode/Voucher number	Collection site	Herbarium	No. of specimens
*X.L. Zhou*	2024MN01–2024MN12	Mianning, Sichuan, China	XJU	12
*X.L. Zhou*	2024NJ01–2024NJ06	Nanjiang, Sichuan, China	XJU	6
*X.L. Zhou*	2024LS01–2024LS05	Longshan, Hunan, China	XJU	5
*X.L. Zhou*	2024SN01–2024SN03	Shennongjia, Hubei, China	XJU	3
*X.L. Zhou*	2024LG01–2024LG06	Langao, Shanxi, China	XJU	6
*Im, H. T.*	01698545	Japan	PE	1
*T.H. Tu*	00021940–00021941	Baoxing, Sichuan, China	PE	2
*Szechuan Econ. Bot. Liangshan Exp.*	00021887	Hunghwa to Wali, Sichuan, China	PE	1
*Y.H. Li*	00021937	Tsenyih, Yunan, China	PE	1
*S. Tschonoski*	00021963	Hakone, Japan	PE	1

### ﻿Morphological comparison

**Figure 3.**
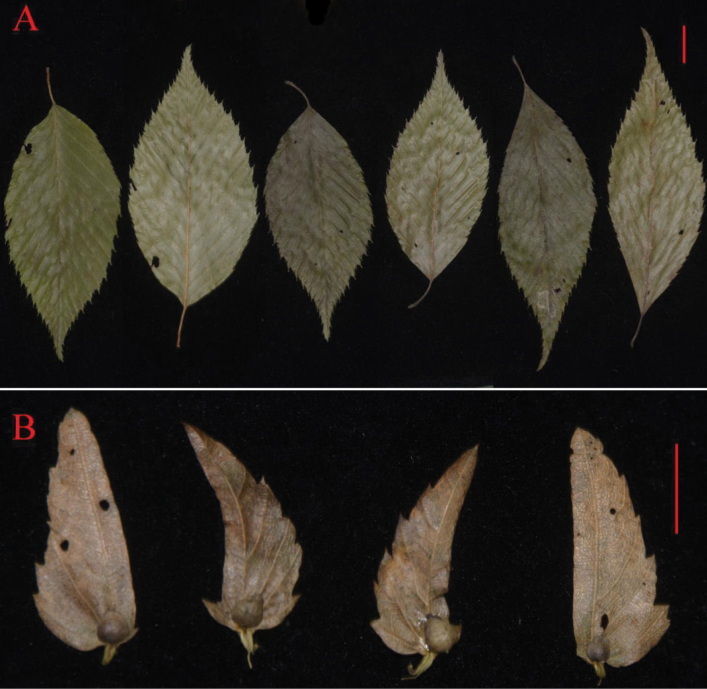
Abundant variations in leaves (**A**) and bracts (**B**) based on the 29 *C.tschonoskii* specimens sharing one identical ITS sequence. Both red bars are one centimeter long.

The newly collected *C.tschonoskii* specimens sharing the identical ITS sequence exhibited substantial morphological variation in basal lobe morphology (inner margin) and leaf characteristics, including length-to-width ratio and presence/absence of the basal lobe (Fig. 3; Tables 1–3). Notably, the morphological variation range based on type specimens of *C.tschonoskii* and its synonyms (*C.falcatibrateata*, *C.obovatifolia*, and *C.paohsingensis*) fell entirely within the variation observed in our newly collected *C.tschonoskii* specimens (Fig. 3; Tables 2, 3). Comparative morphological analysis between the putative new species and *C.tschonoskii* (including type specimens) revealed six consistent, diagnostic differences that corroborate the molecular evidence. The putative new species is characterized by: (1) mucronate-serrate leaf margins (versus setiform-serrate in *C.tschonoskii*); (2) serration restricted to the basal half of outer bract margins (versus serration extending across both basal and apical halves); (3) reduced serration number on outer bract margins (1–3 versus 4–7 serrations per bract); (4) nearly glabrous petioles and annual branchlets (versus sparsely or densely pubescent); (5) longer petioles (1.0–1.7 versus 0.6–1.3 cm); and (6) leaf base shape (rounded, subrounded, cordate, or subcordate versus rounded-cuneate or cuneate) (Table 3). These discrete, non-overlapping morphological characters provide unequivocal evidence for recognizing it as a distinct species.

**Table 3.** Morphological comparison of *C.wenshanensis* and *C.tschonoskii* based on 29 newly collected specimens sharing one identical ITS sequence or all specimens in Table 2. All the specimens in Table 2 and 29 newly collected specimens that share one identical ITS sequence present the same results.

Traits	*Carpinuswenshanensis*	*Carpinustschonoskii*
**LEAF**		
Shape and size	Leaf blade elliptic, oblong, or ovate-lanceolate, 5.4–9.2 × 2.8–4.2 cm, length\width ratio 1.9–2.8; base rounded, subrounded, cordate or subcordate; apex acute, acuminate or caudate, rarely long caudate, 0.4–1.2 cm	Leaf blade elliptic or ovate-lanceolate, 4.5–11.5 × 2.3–5.7 cm, length\width ratio 1.5–2.9; base rounded-cuneate or cuneate; apex acute, acuminate or caudate, 0.3–1.2 cm
Leaf petiole length	**1.0–1.7 cm**	**0.6–1.3 cm**
Petiole pubescent or not	**Nearly glabrous**	**Sparsely or densely pubescen**t
Number of lateral veins on each side	11–14	11–16
Leaf length to number of lateral veins ratio	0.49–0.77	0.36–0.67
Average distance between neighbouring lateral veins	3.8–5.5 mm	3.0–5.7 mm
Leaf margin	**Doubly mucronate-serrate**	**Doubly setiform-serra**te
**ANNUAL BRANCHLET**		
Glabrous or pubescent	**Nearly glabrou**s	**Sparsely or densely pubescent**
**INFRUCTESCENCE**		
Infructescence size	5.5–8.5 × 3.0–4.5 cm	5.0–10.5 × 3.0–5.0 cm
Length of peduncle	1.5–3.0 cm	1.0–4.5 cm
**BRACT**		
Length of bract	2.2–3.1 cm	2.2–3.4 cm
Lobe presence at base of the outer margin	Absence	Absence
Outer margin	Serration only at lower half	Serration at both basal and apical halves
Serration number along the outer bract	**1**–**3**	**4**–**7**
Lobe presence at base of the inner margin	Presence	Absence or presence
Length of inflexed basal lobe (at the base of inner margin)	1.0–2.0 mm	0.0–3.0 mm
**NUTLET**		
Size and shape of nutlet	Broadly ovoid, 4.0–5.0 × 3.5–4.5 mm	Broadly ovoid, 4.0–6.0 × 3.8–5.3 mm
Villous, pubescent or glabrous	Sparsely pubescent and with villous at apex	Sparsely pubescent and with villous at apex
Resinous glandular	Sparsely resinous glandular	Sparsely resinous glandular or none

### ﻿Taxonomic treatment

#### ﻿ Carpinus wenshanensis

Taxon classificationPlantaeFagalesBetulaceae

Xiaolong Zhou sp. nov.

683C10ED-D0B1-5F76-AE83-357D95885DB0

urn:lsid:ipni.org:names:77365616-1

Fig. 1 Chinese name: 文山鹅耳枥

##### Diagnosis.

*Carpinuswenshanensis* is morphologically and phylogenetically most closely allied to *C.tschonoskii*, yet exhibits diagnostic differences in six key characteristics: leaf margin serration (mucronate versus setiform), serrate position on outer bract margin (restricted to the basal half versus extending across both basal and apical halves), fewer serration numbers on outer bract margin (1–3 versus 4–7 serrations per bract), pubescent density of leaf petiole and annual branchlet (nearly glabrous versus sparse or dense), petiole length (1.0–1.7 versus 0.6–1.3 cm), and leaf base morphology (rounded, subrounded, cordate, or subcordate versus rounded-cuneate or cuneate).

##### Holotype.

China • Yunnan: Bozhushan, Wenshan Prefecture, 23°23'02"N, 103°55'50"E, 2500 m alt., 16 August 2024, *X.L. Zhou 2024WS01* (XJU00050376A).

##### Description.

Trees to 11 m tall; bark gray with shallow vertical fissures. Branchlets gray-brown, glabrous, with prominent white to yellowish-white lenticels. Leaves: petiole 1.0–1.7 cm, nearly glabrous; blade elliptic, oblong, or ovate-lanceolate, 5.4–9.2 × 2.8–4.2 cm (length-to-width ratio 1.9–2.8); abaxial surface sparsely villous along veins, bearded in axils of lateral veins, adaxial surface glabrous; base rounded, subrounded, cordate, or subcordate; apex acute, acuminate, or caudate; margin irregularly or doubly mucronate-serrate; lateral veins 11–14 pairs. Inflorescences 5.5–8.5 × 3.0–4.5 cm; peduncle 1.5–3.0 cm, sparsely villous. Bracts semiovate- to ovate-lanceolate, loosely imbricate, 2.2–3.1 × 0.7–1.0 cm, adaxially sparsely pubescent; outer margin with 1–3 serrations restricted to the basal half, lacking basal lobe; inner margin entire, bearing an inflexed basal lobe (1.0–2.0 mm); apex acute or acuminate; primary veins 3–4 (excluding inflexed lobe), venation prominent. Nutlets broadly ovoid, 4.0–5.0 × 3.5–4.5 mm, sparsely to densely pubescent, villous at apex, sparsely resinous-glandular, prominently ribbed.

##### Etymology.

To date, this hornbeam is known only from a single population in Wenshan Prefecture. Given its apparently restricted endemic status and limited distribution, we have chosen the specific epithet *wenshanensis* to reflect its geographic origin.

##### Phenology.

Flowering time unknown, and fruiting time from June to September.

##### Habitat, distribution and conservation.

During a six-day survey of Bozhushan and adjacent areas in Wenshan Prefecture, we documented hornbeam distributions but failed to locate the additional population of *C.wenshanensis*. This newly described species occupies higher elevations than sympatric congeners (e.g., *C.fargesii*). The sole known population comprises a single reproductive adult (11 m tall) and three seedlings (0.5–2.5 m) in a roadside secondary forest. Notably, the mature tree produces abundant fruit (Fig. 1), suggesting either: (1) self-pollination capability, or (2) pollen contribution from undetected conspecifics. This reproductive ecology requires further study. Following IUCN Red List criteria (2016), we recommend classifying *C.wenshanensis* as Critically Endangered (CR) due to extremely limited distribution (single location) and minimal population size (1 reproductive individual). However, its occurrence within a protected area offers some conservation assurance. Expanded surveys are needed to assess whether additional populations exist.

##### Additional specimens examined for this new hornbeam species.

China • Yunnan: Wenshan Prefecture, 23°23'02"N, 103°55'50"E, 2500 m alt., 16 August 2024, *X. L. Zhou 2024WS02* (XJU00050376B) and *X. L. Zhou 2024WS03* (XJU00050376C).

## ﻿Discussion

The presence of a basal lobe on the inner bract margin represents a key diagnostic feature for species delineation in *Carpinus* (Li and Skvortsov 1999; Lu et al. 2017; Lu 2020). Our study identifies 14 species exhibiting this trait, including *C.putoensis*, *C.langaoensis*, *C.kweichowensis*, *C.tschonoskii* (partially lacking lobes; Fig. 3; Table 3), *C.tientaiensis*, *C.mianningensis*, *C.viminea*, *C.fargesii*, *C.londoniana*, *C.laxiflora*, *C.betulus*, *C.austrobalcanica*, *C.tropicalis*, and *C.caroliniana* (Holstein and Weigend 2017; Lu et al. 2017, 2024; Kuzmanović et al. 2024). Among these, only nine lobed-bract (inner bract margin) species occur in China (*C.putoensis*, *C.langaoensis*, *C.kweichowensis*, *C.tschonoskii*, *C.tientaiensis*, *C.mianningensis*, *C.viminea*, *C.fargesii*, and *C.londoniana*), where they are geographically isolated from others lobed-bract species. For example, *C.laxiflora* is restricted to Japan and the Korean Peninsula (Lu et al. 2024), and *C.caroliniana*, *C.tropicalis*, *C.betulus*, and *C.austrobalcanica* are distributed in North America, Europe, and/or West Asia (Furlow 1987; Kuzmanović et al. 2024). Phylogenetic analysis of ITS sequence variations corroborates the distinctiveness of these lobed-bract (inner margin) species from non-lobed congeners, and such lobed-bract species in China from those in other countries (Lu et al. 2017, 2018, 2024; Dong et al. 2022; Kuzmanović et al. 2024; Fig. 2). These findings confirm the basal lobe’s taxonomic significance and highlight regional divergence in related lobed-bract species between China and other areas.

While *C.tropicalis*, and *C.kweichowensis* were excluded from phylogenetic analyses due to unavailable sequence data (Kuzmanović et al. 2024; Fig. 2), morphological and biogeographic evidence supports their close affinities: *C.tropicalis* with *C.caroliniana*, and *C.kweichowensis* with *C.mianningensis*/*C.putoensis* (Furlow 1987; Yi 1992; Li and Skvortsov 1999; Lu et al. 2017; Kuzmanović et al. 2024). Notably, *C.tropicalis* together with *C.caroliniana*, classified as a complex, are characterized by prominent lobes at bases of both inner and outer bract margin (Furlow,1987), contrasting with the putative new species (Fig. 2). Molecular data corroborate the morphologically inferred relationship between *C.mianningensis* and *C.putoensis* (Fig. 2), supporting their grouping with *C.kweichowensis* based on shared traits.

Despite historical recognition of *C.falcatibrateata*, *C.obovatifolia*, and *C.paohsingensis* as distinct species (Li and Skvortsov 1999; Lu et al. 2017), comparative morphological analysis of type specimens (PE 00021940, PE 00021941, PE 00021937, PE 00021887) reveals their variation falls entirely within the range of *C.tschonoskii*, as demonstrated by 29 specimens sharing one identical ITS sequence (Fig. 3, Tables 1–3). Although *C.paohsingensis* was maintained as independent species status by Govaerts and Frodin (1998), we recommend synonymizing all three taxa under *C.tschonoskii*. Therefore, an integrative assessment of molecular, morphological, and geographic data indicates that the putative new species is most closely related to *C.tschonoskii*.

Despite their sister relationship, the putative new species exhibits significant divergence from *C.tschonoskii* in both molecular and morphological traits. Molecular analyses reveal three fixed nucleotide differences in ITS sequences (Fig. 3, Table 1), providing clear genetic differentiation between the putative new species and *C.tschonoskii*. Morphologically, it differs by its mucronate-serrate leaf margins (versus setiform-serrate in *C.tschonoskii*), outer bract margins serrate only on the basal half (versus both halves), fewer outer bract serrations (1–3 versus 4–7 serrations per bract), nearly glabrous petioles and branchlets (versus sparsely or pubescent), longer petioles (1.0–1.7 versus 0.6–1.3 cm), and leaf base shape (rounded, subrounded, cordate, or subcordate versus rounded-cuneate or cuneate). Under an integrative species delimitation frame (Lu et al. 2021, 2024), these consistent, non-overlapping differences in both DNA sequences and morphological features support the recognition of this small Wenshan population as a distinct species.

## Supplementary Material

XML Treatment for Carpinus wenshanensis

## ﻿Additional information

### Author ORCIDs

Xiao-Long Zhou https://orcid.org/0009-0009-4372-247X

### Data availability

All of the data that support the findings of this study are available in the main text or Supplementary Information.

## Supplementary materials

Supplementary material 1 *C.wenshanensis*-ITS sequences Data type txt https://binary.pensoft.net/file/1374524This dataset is made available under the Open Database License (http://opendatacommons.org/licenses/odbl/1.0/). The Open Database License (ODbL) is a license agreement intended to allow users to freely share, modify, and use this Dataset while maintaining this same freedom for others, provided that the original source and author(s) are credited.Xiao-Long Zhou, Qian-Na An
